# Differential expression of senescence tumour markers and its implications on survival outcomes of breast cancer patients

**DOI:** 10.1371/journal.pone.0214604

**Published:** 2019-04-18

**Authors:** Rahmawati Pare, Patsy S. Soon, Aashit Shah, Cheok Soon Lee

**Affiliations:** 1 Department of Biomedical Science and Therapeutic, Faculty of Medicine & Health Sciences, Universiti Malaysia Sabah, Kota Kinabalu, Sabah Malaysia; 2 Ingham Institute for Applied Medical Research, Liverpool, NSW Australia; 3 Discipline of Pathology, School of Medicine, Western Sydney University, Liverpool, NSW Australia; 4 Breast Surgery Unit, Bankstown Hospital, Bankstown, NSW Australia; 5 South Western Sydney Clinical School, University of New South Wales, Liverpool, NSW Australia; 6 Breast Surgery Unit, Liverpool Hospital, Liverpool, NSW Australia; 7 Department of Anatomical Pathology, Liverpool Hospital, Liverpool, NSW Australia; 8 Central Clinical School, Faculty of Medicine and Health, University of Sydney, Camperdown, NSW Australia; University of South Alabama Mitchell Cancer Institute, UNITED STATES

## Abstract

Breast cancer is a heterogeneous disease displaying different histopathological characteristics, molecular profiling and clinical behavior. This study describes the expression patterns of senescence markers P53, DEC1 and DCR2 and assesses their significance on patient survival as a single or combined marker with P16 or P14 using breast cancer progression series. One thousand and eighty (1080) patients with primary invasive ductal carcinoma, no special type, were recruited through an 11-year retrospective study period. We constructed tissue microarrays of normal, benign hyperplasia, ductal carcinoma in situ and invasive ductal carcinoma from each patient and performed immunohistochemical staining to study the protein expression. Statistical analysis includes Pearson chi-square, Kaplan-Meier log ran test and Cox proportional hazard regression were undertaken to determine the associations and predict the survival outcomes. P53, DEC1 and DCR2 expression correlated significantly with normal, benign, premalignant and malignant tissues with (p<0.05). The expression profile of these genes increases from normal to benign to premalignant and plateaued from premalignant to malignant phenotype. There is a significant association between P53 protein expression and age, grade, staging, lymphovascular invasion, estrogen receptor, progesterone receptor and HER2 whereas DCR2 protein expression significantly correlated with tumour grade, hormone receptors status and HER2 (p<0.05 respectively). P53 overexpression correlated with increased risk of relapse (p = 0.002) specifically in patients who did not receive hormone therapy (p = 0.005) or chemotherapy (p<0.0001). The combination of P53+/P16+ is significantly correlated with poor overall and disease-free survival, whereas a combination of P53+/P14+ is associated with worse outcome in disease-free survival (p<0.05 respectively). P53 overexpression appears to be a univariate predictor of poor disease-free survival. The expression profiles of DEC1 and DCR2 do not appear to correlate with patient survival outcomes. The combination of P53 with P16, rather P53 expression alone, appears to provide more useful clinical information on patient survival outcomes in breast cancer.

## Introduction

Breast cancer is the commonest malignancy in women worldwide and is a highly heterogeneous cancer. Increased screening programme and awareness has had significant impact on breast cancer survival rate. This is due to the early detection of breast cancer that has allowed early effective treatment [1]. However, there is still a need to understand the fundamental aspect of breast tumourigenesis.

Senescence-associated markers are found to play an important role in the development of a wide range of human cancers. Latest findings have indicated the role of senescence markers in cancer progression which showed increased expression in premalignant but lost in malignant lesions [2]. Such is the interest in cellular senescence in tumourigenesis that it has been suggested that a ‘senescence index’ may be used as a prognostic indicator and that the use of oncogene-induced senescence markers in the clinic could be useful in detecting cancer at early stage disease, and the loss of these markers would be indicative of tumour progression to a malignant stage.

A number of prognostic and predictive factors have been used in the management of cancer patients. P53 protein expression is one of the tumour markers that have been thoroughly investigated; however, its value in helping breast cancer management has not been universally accepted. P53 protein has been described since 1979 as a virus-associated tumour antigen, an oncogene and finally an important tumour suppressor [3]. Physiologically, wild-type P53 is a tumour suppressor gene that mediates cell cycle regulatory pathways either via cell cycle arrest, apoptosis or cellular senescence [4]. Abnormalities of the gene may give rise to a cancer after accumulation of many mutations in the same cells [5].

DEC1 is a basic helix loop-helix (BHLHB2) transcription factor that can be triggered by numerous extracellular stimuli such as growth factors, serum starvation, hypoxia, hormones, nutrient, cytokines, light and infection. It is involved in a wide range of signaling pathways including development, cell differentiation, cell growth, cell death, oncogenesis, immune systems, circadian rhythm, and homeostasis [6]. On the other hand, DCR2, also known as TRAIL-R4, is a truncated receptor for tumor necrosis factor (TNF)-related apoptosis-inducing ligand (TRAIL or Apo2L). This protein is well known for its anti-apoptotic activity by inhibiting the ligand formation between TRAIL and the death receptors especially DR5 [7]. Of the putative functions of DEC1 and DCR2, both de-novo markers are recently found to be candidate genes for premature cellular senescence [8]. Both DEC1 and DCR2 are target genes of P53 in the induction of premature senescence.

The cellular senescence pathway is a cellular protective mechanism against mutations by irreversibly arresting cell growth. In contrast to apoptosis, senescent cells are still metabolically active despite its permanent cytostatic phase. Biologically, there are two subtypes of senescence which are replicative and oncogene-induced senescence. Replicative senescence mainly involves progressive shortening of telomere whereas oncogene-induced senescence can be triggered by activated RAS oncogene, DNA damage or other cellular stresses via two well established pathways; namely p16 INK4a –Rb and ARF-P53-p21 [9].

In this study, our interest is focused on the P53, DEC1 and DCR2 senescence marker characteristics and its importance in predicting breast cancer patient survival as a single prognostic factor or when in combination with other senescence markers. Combination with P14 and P16 were conducted as these two markers were found to play important roles in breast tumorigenesis in our previous study [10]. We hypothesize that expression of these proteins increases as the lesion progress but is lost in malignant tumours, and the combined effect with other markers may provide more statistically significant prognostic information.

## Materials and methods

### Patients

The cohort used in this study has been reported previously [10]. It consists of 1080 female breast cancer patients from the South Western region of Sydney. An 11-year retrospective cohort of patients diagnosed with invasive ductal carcinoma (IDC) were included and excludes those who had pre-operative chemotherapy or recurrent disease at the time of diagnosis. The project complies with the ethical requirements of the Human Research Ethics Committee of the South Western Sydney Local Health District Ethics and Research Governance Office (HREC/12/LPOOL/158), and guidelines from the National Health and Medical Research Council of Australia. Patients’ demographic, clinicopathological and follow up information are obtained from the electronic medical records that include the pathology and medical oncology databases (data has been published [10]). For all patients, the following data are annotated: age at diagnosis, tumour size, tumour grade, disease stage, presence or absence of lymphovascular invasion (LVI) and lymph node involvement (LNI). The end of follow-up period was at 30 June 2014 with median follow-up overall survival of 4.96 years (0.15–14.86 years) and disease-free survival of 4.76 years (0.11–13.84 years).

### Tissue microarray construction

Resected tumour tissues at surgery are fixed in 10% buffered formalin at 45°C. The specimens are fixed for no longer than 12 hours. All fixed tissues then send to the Department of Anatomical Pathology, South Western Sydney Area Pathology Service, Liverpool Hospital (New South Wales, Australia) for histopathology processing. After fixation, tissue is dehydrated through a series of graded ethanol, immersed into the xylene and then infiltrated with paraffin. The blocks are cut at 4μm thin sections and subjected to H&E staining. The H&E slide of every case was reviewed along with the original histopathology reports. Representative areas of interest were marked on donor H&E sections to assist in obtaining suitable areas for incorporation into the arrays. Duplicate 1.0mm cores of normal, atypical ductal hyperplasia, DCIS and IDC tissues per patient were constructed into tissue array. 51.2% atypical ductal hyperplasia and 72.0% DCIS were concurrently present with the corresponding normal and malignant tumour tissues. Arrays sections with 4–5 μm thickness were mounted on Superfrost ultra plus glass slides.

### Immunohistochemistry

Prior to immunohistochemistry staining, the tissue microarray sections were incubated in the oven at 60°C for at least 1 hour. Deparaffinisation process includes immersion in xylene three times followed by rehydration through graded, decreasing concentrations of ethanol ending in running water. The sections were then incubated in the pre-heated 98°C citrate buffer, pH 6.0, in hot water bath for 20 minutes followed by cooling at room temperature for 20 min. Antigen retrieval of DEC1 and DCR2 were conducted in Tris/EDTA pH9. Endogenous peroxidase was blocked with hydrogen peroxidase for 20 minutes before antibody incubation. Antibody was then subjected to slides for 20 minutes at room temperature in a moist chamber (P53, 1:800 dilution; Invitrogen, CA, USA. DEC1, 1:25 dilution; Novus Biological, CO, USA. DCR2, 1:100 dilution; Abcam, MA, USA). Goat secondary antibody was added for 15 minutes to detect any primary antibody-antigen complex, to which high sensitivity 3, 3’-diaminobenzidine tetrahydrochloride is then added, followed by hematoxylin counterstaining and mounted.

#### Scoring of immunohistochemically stained sections

Assessment of P53 immunoreactivity is based on established method. Protein expressions are analysed semi-quantitatively by estimating the percentage of immunoreactivity in the nucleus and disregarding the cytoplasmic staining. Two independent pathologists who were blinded to the patients’ details were initially trained to assess a test series of at least 36 tissue core sections with a multiheader microscope to ensure consistent and reliable interpretation. Intra- and inter-observer agreement were estimated using Kappa (κ) and Spearman rho (ρ). Training was ended when the desired level of agreement, consistent over time, was achieved (κ>0.6 and ρ>0.8). An average score was obtained from the duplicate cores of each tissue sample. Score of more than or equal to 10% were considered to have an increase in expression [11–14]. For DEC1 and DCR2, the intensity and distribution of the immunoreactivity were recorded. DEC1 was scored according to the study by Xu et al. [15]: intensity score (0, negative; 1, weak; 2, moderate; or 3, strong) was multiplied to distribution score (0, <5%; 1, 5–25%; 2, 26–50%; 3, 51–75% or 4, >75%). Final score ranging from 0–12 classified into negative (0–4), weakly positive (5–8) and strongly positive (9–12). Scoring for DCR2 was performed and adapted as described by Sanlioglu et al[16]: intensity (0, negative; 1, weak; 2, moderate or 3, strong) was added to distribution (0, <10%; 1, 10–40%; 2, 40–70% or 3, >70%) giving final score as negative (≤1) or positive (≥2).

### Statistical analyses

Statistical analyses were executed using the IBM SPSS Statistics 23.0 programme. The parameters were categorized to assist the analyses. P values of <0.05 were regarded as statistically significant. Pearson chi-square test was performed to analyse the associations between expression of each of the proteins and clinicopathological data. Univariate survival analyses were conducted using the Kaplan-Meier Log-rank test and simple cox regression. The effects of multiple covariates on survival were examined using multivariable Cox Proportional Hazards regression test. Variables were selected if the univariate p-value ≤0.2 to be included in multivariate model. The proportional hazards assumption was checked with hazards functions plot, log-minus-log plot and Schoenfeld residuals plot. The assumption of hazards ratio is constant over time and was not violated. The clinical endpoints for overall survival (OS) defined as time from date of surgery until death by any cause and disease-free survival (DFS) defined as time from date of surgery until any recurrence (locoregional and/or distant, whichever came first), were calculated based on the documented dates of death, recurrence or last follow up. Patients who were still alive (for OS) or had no disease recurrence (for DFS) at last follow up were censored. Missing data were not treated.

## Results

### Patients

All 1080 female patients were newly diagnosed breast cancer cases with age ranging from 27 to 102 years (median 59).

### Expression of senescence markers

**Fig 1:**
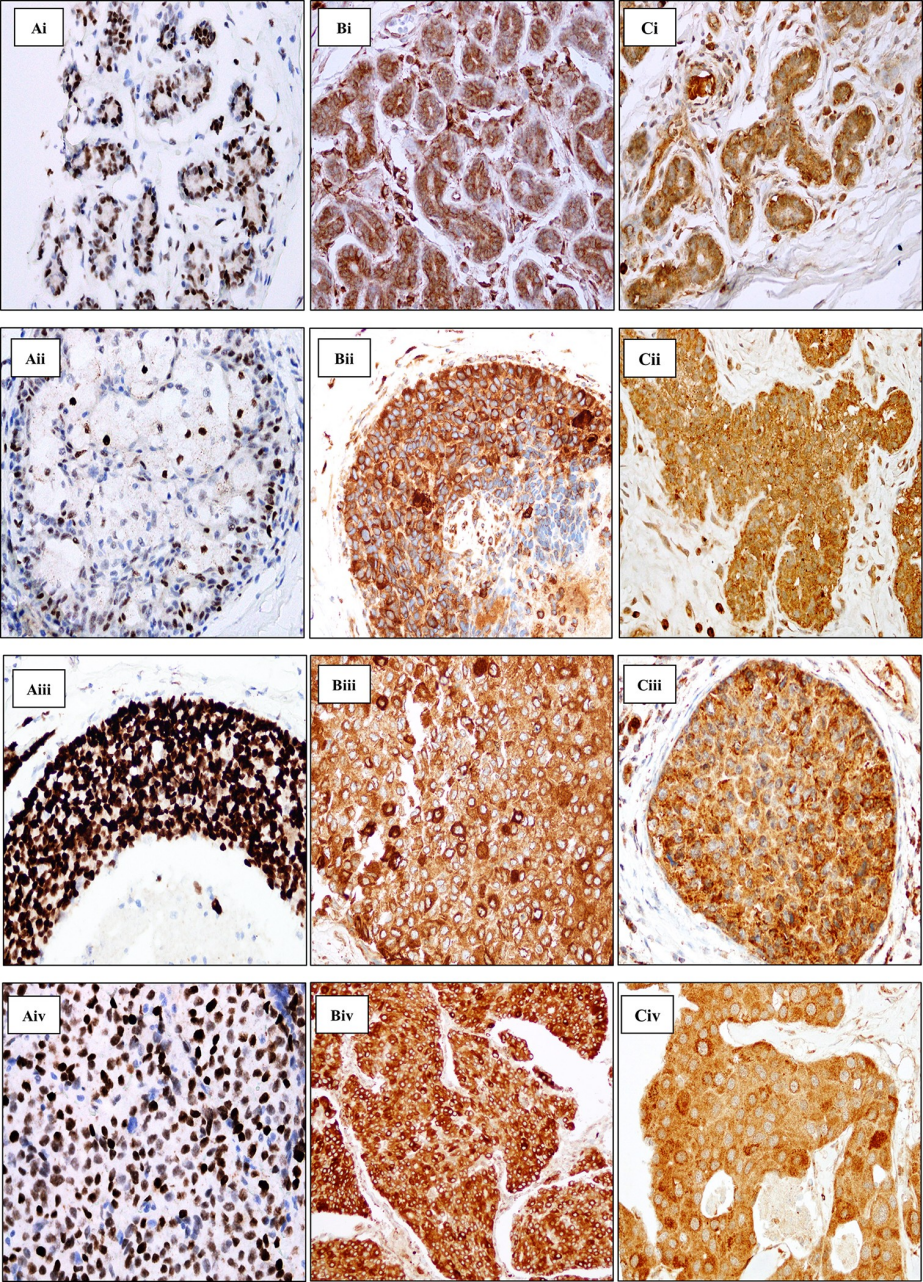
**Immunohistochemical staining of P53 (A), DCR2 (B) and DEC1(C).** Ai-Aiv shows clear and distinct nuclear staining of P53 in the respective tissues. Bi-Biv shows DCR2 immunoreactivity in the cytoplasm with minor protein expression masking over the nucleus in the respective tissues. Ci-Civ shows DEC1 moderate and strong granular staining of the cytoplasm in the respective tissues. i: Normal tissue, ii: Atypical Ductal Hyperplasia (ADH), iii: Ductal Carcinoma in situ (DCIS), iv: Invasive ductal carcinoma (IDC). Magnification x400.

P53 protein expression is predominant in the nucleus and incidental faint cytoplasmic staining is seen. If present, the immunoreactivity of P53 in normal and benign tissues generally presented as weak nuclear staining (Fig 1 Ai & Aii). The signal increases as the lesion progresses to premalignant and malignant features (Fig 1 Aiii & Aiv). Generally, the expression of P53 appears to increase from normal to ADH to DCIS, and then slightly decreases from DCIS to IDC. In the present study, we found the matching normal tissue that is positively stained is more likely to be further positively expressed in more advanced lesions. However, P53 is more likely to be reduced in expression in IDC (p = 0.032). DCIS are more likely to have greater P53 expression than IDC lesions (p<0.0001) (Table 1).

**Table 1:** Association of protein expression with breast cancer progression.

	P53 Expression			DCR2 Expression			DEC1 Nuclear Expression			
	Low	High	p-value	Negative	Positive	p-value	Negative	Weak	Strong	p-value
	(<10%)	(≥10%)		(<10%)	(≥10%)		(0–4)	(5–8)	(9–12)	
Normal	419	14	**<0.0001**^**a**^	371	567	**<0.0001**^**a**^	91	869	1	**<0.0001**^**a**^
	(86.4%)	(2.9%)		(39.6%)	(60.4%)		(9.5%)	(90.4%)	(0.1%)	
ADH*	266	24	**<0.0001**^**b**^	91	400	**<0.0001**^**b**^	43	455	3	**<0.0001**^**b**^
	(66.5%)	(6.0%)		(18.5%)	(81.5%)		(8.6%)	(90.8%)	(0.6%)	
DCIS^†^	482	152	**<0.0001**^**c**^	53	650	**<0.0001**^**c**^	79	654	11	**<0.0001**^**c**^
	(65.7%)	(20.7%)		(7.5%)	(92.5%)		(10.6%)	(87.9%)	(1.5%)	
IDC^‡^	673	22	**0.032**^**d**^	63	969	**<0.0001**^**d**^	125	912	13	**<0.0001**^**d**^
	(69.5%)	(2.3%)		(6.1%)	(93.9%)		(11.9%)	(86.9%)	(1.2%)	

Chi square p-value indicates the differences between:

(a) Normal vs. ADH

(b) ADH vs. DCIS

(c) DCIS vs. IDC and

(d) IDC vs. Normal. Percentage represent ratio of positive or negative to the total number of cases.

*ADH: Atypical Ductal Hyperplasia

†DCIS: Ductal Carcinoma in situ

‡IDC: Invasive Ductal Carcinoma

DCR2 is seen primarily in the cytoplasmic region of the breast epithelium (Fig 1 Bi). Immunostaining of DCR2 is characterized by variable staining intensity but a higher degree of distribution can be seen in the tissues progressing from early development to malignant lesion. Occasionally, a few cells have strong staining in the cytoplasm compared to the adjacent cells which may form a ring shape of concentrated dark brown around the nuclear region. Intense cytoplasmic staining is sometimes seen to mask the nuclear compartment (Fig 1 Bii—Biv). Positive-stained cells in the precursor lesions are more likely to continue to overexpress the protein in the more advanced lesion. The association of DCR2 expression in each lesion is statistically significant (p<0.0001) (Table 1).

DEC1 expression is localised in both the nucleus and cytoplasm. The staining is uniformly homogenous in normal, benign, premalignant and malignant breast tissues (Fig 1 Ci–Civ). Most of the breast tissues have weak activation of the DEC1 gene. The strong activation appears to increase from normal to benign to premalignant lesions, and then decreases from premalignant to malignant tissues in both the nucleus and cytoplasm; although the percentage of staining is small. The association of DEC1 expression between each breast lesion appears to be statistically significant (p<0.0001) (Table 1).

### Association with clinicopathological parameters

The significance of P53 protein expression in breast tumour was assessed in relation to standard prognostic factors. We found P53 protein expression was significantly associated with age, grade, staging, lymphovascular invasion (LVI), estrogen receptor (ER), progesterone receptor (PR) and HER2 (Table 2). However, the marker did not significantly associate with tumour size and lymph node metastases (LNM). P53 downregulation was associated with good prognostic indicators such as moderate differentiation, early stage tumour, absence of lymphovascular invasion, positive ER and PR as well as negative HER2. However, P53 downregulation was associated with older age.

**Table 2:** Association of senescence-associated markers with clinicopathological variables.

		P53 protein Expression				DCR2 protein Expression			
		Low	High	χ^2^	p	Low	High	χ^2^	p
		(<10%)	(≥10%)			(<10%)	(≥10%)		
Age	≤60 years	381	178	14.360	<0.0001*	38	509	1.441	0.230
	>60 years	388	106			25	460		
Tumour size	≤20mm	468	157	2.674	0.102	36	574	0.107	0.743
	>20mm	301	127			27	395		
Grade	Well	238	19	124.019	<0.0001*	15	234	8.964	0.011*
	Moderate	333	95			16	408		
	Poor	198	170			32	327		
Stage	I	341	100	7.980	0.046*	22	407	1.972	0.578
	II	338	151			33	449		
	III	83	30			8	104		
	IV	7	3			0	9		
LVI	Absent	528	163	11.669	0.001*	40	633	0.088	0.767
	Present	241	121			23	336		
LNI	Absent	494	170	1.708	0.191	33	612	2.931	0.087
	Present	275	114			30	357		
ER	Negative	107	114	88.636	<0.0001*	28	187	21.700	<0.0001*
	Positive	621	153			33	730		
PR	Negative	188	132	50.124	<0.0001*	29	281	7.578	0.006*
	Positive	541	135			32	637		
HER2	Negative	404	141	14.352	<0.0001*	31	498	6.085	0.014*
	Positive	58	46			13	90		

* Statistically significant p<0.05

We found overexpression of DCR2 protein was associated with unfavourable prognostic factors such as poor tumour differentiation, ER and PR negativity and HER2 positivity (Table 2). There were no significant associations made with other prognostic indicators such as age, tumour size, staging, lymphovascular invasion and lymph node involvement. Both nuclear and cytoplasmic immunoreactivity of DEC1 were not significantly associated with all of the clinicopathological data.

### Survival analyses

The differences in overall survival and disease-free survival for patients with different levels of P53 expression was analysed using Kaplan-Meier log rank test. In the P53 negative group, a total of 105 patients had died (n = 735, 14.29%) while 630 patients were still alive (n = 735, 85.71%) during the follow up period. The 3-year, 5-year and 10-year survival rate was 94.0%, 87.5% and 75.6% respectively. The positive P53 group had 42 deaths (n = 269, 15.61%) with 227 patients still alive (n = 269, 84.39%) during the follow up period. The 3-year, 5-year and 10-year survival rate was 89.4%, 84.0% and 69.4% respectively.

**Fig 2:**
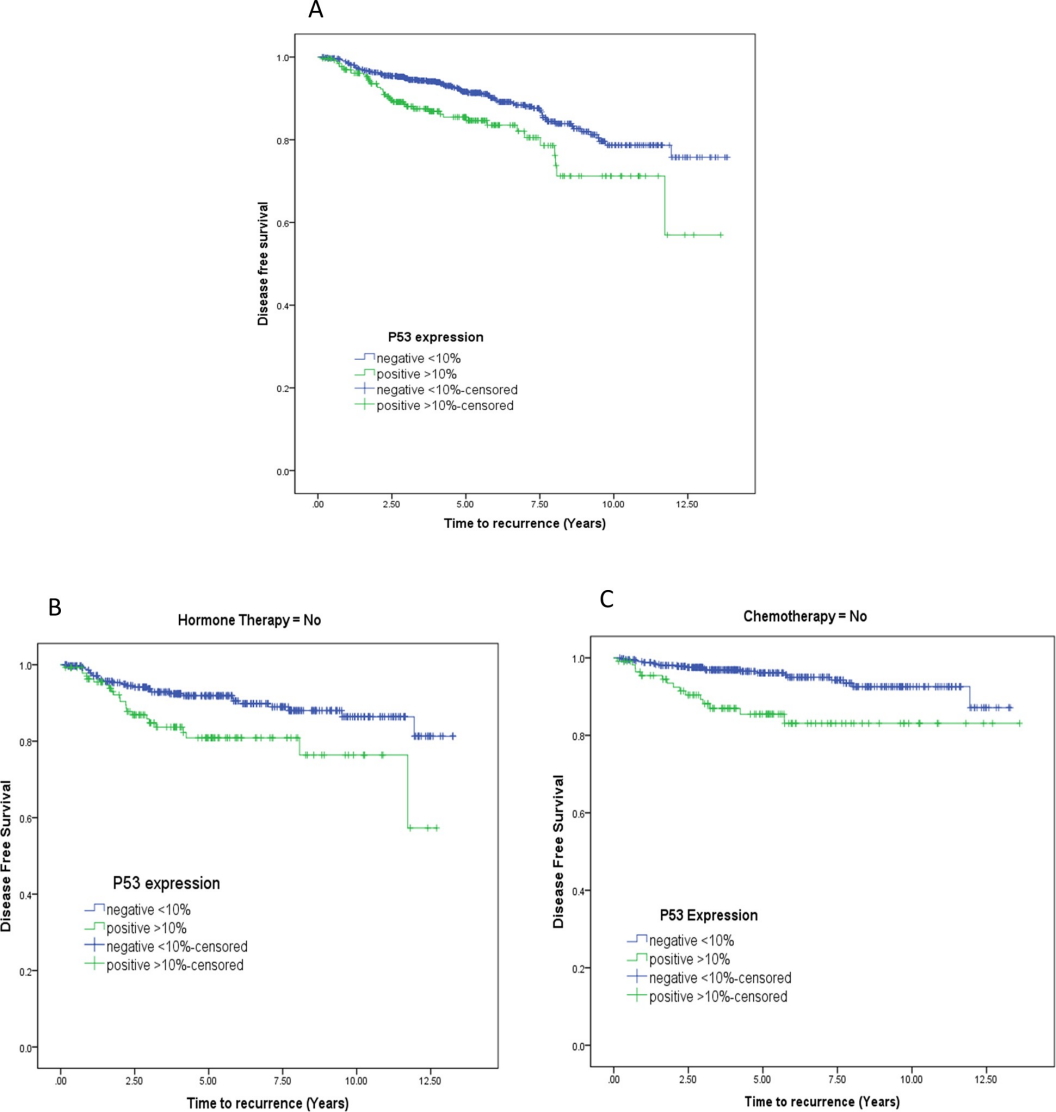
Survival probability based on P53 marker. **A**: Disease free survival probability (Kaplan-Meier curve, negative vs. positive, p = 0.002) **B**: Disease-free survival for hormone therapy (P = 0.005).**C**: Disease-free survival for chemotherapy (p<0.0001).

There was a total of 119 tumour relapse cases consisting of 78 P53 negative cases and 41 P53 positive cases. Recurrence rate for the P53 negative group was 10.70% (n = 729, event = 78) and 15.36% (n = 267, event = 41) for the P53 positive group. Univariate cox regression analysis showed P53 was a significant prognostic factor in predicting disease free survival (HR 1.805 95% CI 1.23–2.639 p = 0.002) (Fig 2A) but not overall survival. P53 protein overexpression is correlated with increased risk in developing the disease later especially in the patient not receiving hormone therapy (p = 0.005) (Fig 2B) or chemotherapy (p<0.0001) (Fig 2C).

While the DCR2 positive group showed a pattern of worse survival compared to negative group, it was not statistically significant (log rank test, p = 0.765). We also found no significant differences in survival analysis of patient groups with differential expression of DEC1.

Multivariable analysis was conducted used the Multiple Cox regression. Variables with univariate p-value of ≤0.2 were included in the multivariable analysis. However, when multivariate analysis incorporating the clinicopathology parameters was conducted, P53 did not show any significant findings for both overall and disease-free survival (Table 3).

**Table 3:** Univariate and multivariate cox regression to estimate the hazards ratio.

Variable	Univariate analysis			Multivariate analysis		
	RR	95% CI	P-value	RR	95% CI	P-value
**Overall survival (OS)**						
Age	1.367	0.992–1.883	0.056	1.608	0.958–2.698	0.072
Tumour size	1.892	1.373–2.607	<0.0001*	0.778	0.403–1.502	0.455
Grade	2.368	1.485–3.775	<0.0001*	0.855	0.360–2.029	0.722
Staging	16.302	7.523–35.327	<0.0001*	24.939	6.008–103.513	<0.0001*
LVI	3.143	2.262–4.368	<0.0001*	2.079	1.156–3.738	0.015*
LNI	2.429	1.758–3.354	<0.0001*	0.790	0.387–1.614	0.518
ER	0.377	0.270–0.527	<0.0001*	0.313	0.137–0.716	0.006*
PR	0.399	0.288–0.554	<0.0001*	1.124	0.514–2.459	0.769
HER2	1.382	0.765–2.496	0.283	0.718	0.370–1.396	0.329
P53	1.329	0.928–1.902	0.121	0.604	0.335–1.091	0.095
DEC1	1.194	0.275–5.174	0.813			
DCR2	0.902	0.459–1.772	0.765			
**Disease-free survival (DFS)**						
Age	0.815	0.568–1.170	0.267	1.106	0.609–2.007	0.741
Tumour size	1.975	1.379–2.829	<0.0001*	0.870	0.396–1.909	0.728
Grade	4.093	2.246–7.460	<0.0001*	3.030	0.925–9.930	0.067
Staging	12.790	4.922–33.238	<0.0001*	6.697	1.355–33.096	0.020*
LVI	4.003	2.747–5.833	<0.0001*	2.694	1.362–5.326	0.004*
LNI	2.798	1.945–4.024	<0.0001*	1.255	0.513–3.069	0.619
ER	0.293	0.200–0.427	<0.0001*	0.517	0.214–1.245	0.141
PR	0.432	0.297–0.629	<0.0001*	0.837	0.355–1.975	0.685
HER2	1.649	0.859–3.167	0.133	0.748	0.359–1.559	0.439
P53	1.805	1.23–2.639	0.002*	0.675	0.352–1.295	0.237
DEC1	0.831	0.108–6.399	0.859			
DCR2	0.905	0.422–1.944	0.799			

Multivariate is the step to see multiple independent risk factor collective effect on breast cancer patient survival.

* Statistically significant p<0.05

### Combined effect of P53 on survival outcomes

Combined effect was analysed if the marker is significant in predicting breast cancer patient survival as a single prognostic factor. P53 was assessed with the other two important senescence-associated markers P16 and P14. Data regarding P14 and P16 on this cohort has been published previously [10]. Both markers are involved in the initiation of senescence pathway and encoded in the same gene of INK4A/ARF.

#### i) Effect of combining P53 and P14 expression

**Fig 3:**
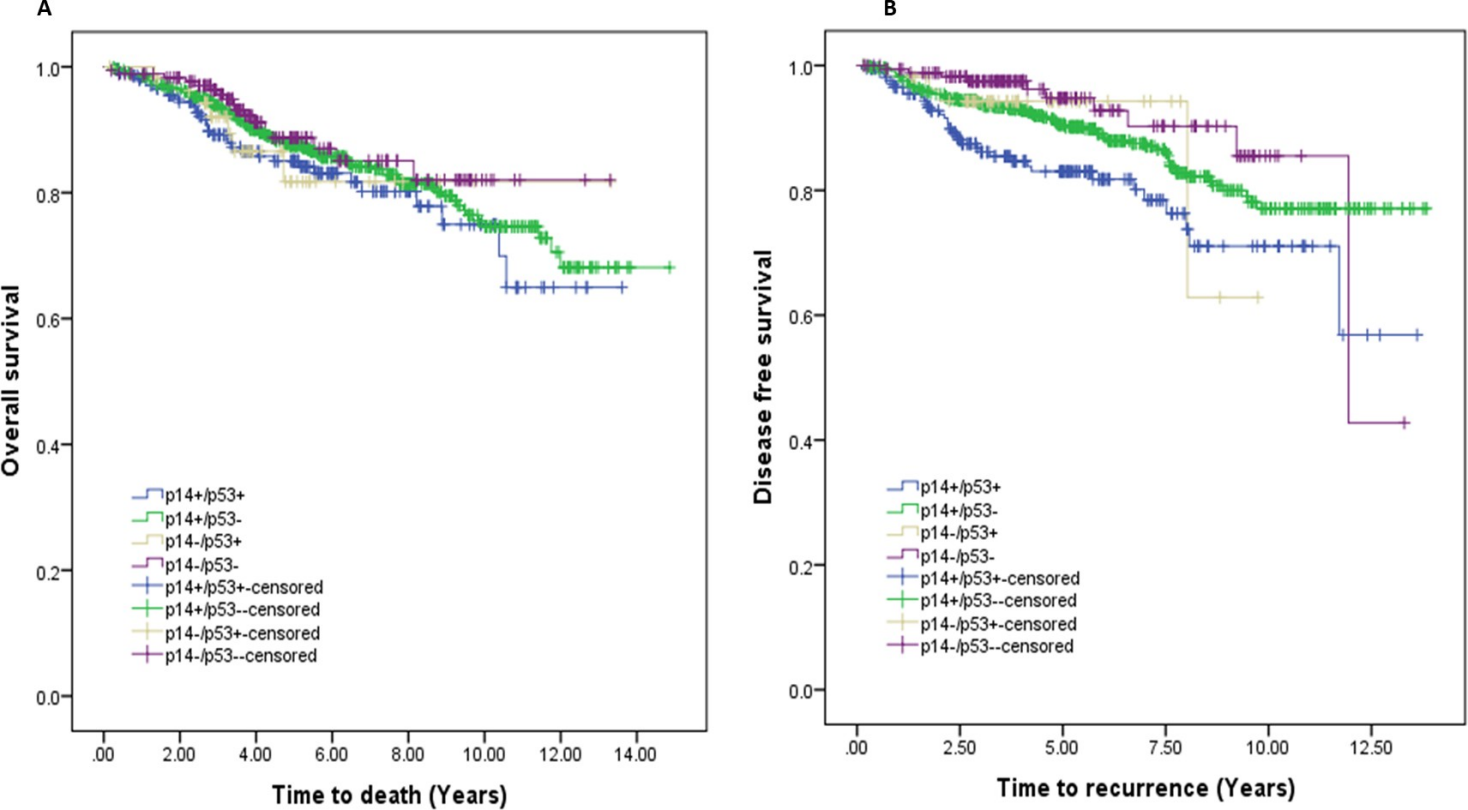
Survival probability based on joint effect of P14 and P53 expression. **A:** Overall survival, no significant difference was seen between each combination **B:** Disease free survival probability based on joint effect of P14 and P53 expression. P14+/P53+ VS. P14-/P53- (p = 0.001) and P14+/P53+ vs. P14+/P53- (p = 0.007) showed significant differences in DFS. The worst outcome was seen in patients with P14+/P53+ tumours.

The effects of combining P14 and P53 expression on patient’s outcome were analysed using univariable models. Combination of P14 and P53 showed significant differences in patient’s disease-free survival only (Fig 3). The worst outcome was seen in patients with P14+/P53+ tumours. Relative to patients with P14 and P53 negative cancers, patients with breast cancers displaying strong P14 and P53 expressions had an adjusted 3-fold increased risk of having disease recurrence (HR = 3.103, 95% CI: 1.539–6.256) (Table 4).

**Table 4:** Association of p14 and P53 status with overall and disease-free survival.

	Time to death			Time to recurrence		
	HR	95% CI	p-value†	HR	95% CI	p-value†
a) Joint effect						
P14+/P53+	1.595	0.891–2.858	0.116	3.103	1.539–6.256	0.002*
P14+/P53-	1.263	0.749–2.129	0.382	1.759	0.904–3.423	0.096
P14-/P53+	1.505	0.624–3.633	0.363	1.495	0.468–4.771	0.497
P14-/P53-	reference			Reference		
b) Stratified effect						
P14+/P53+	1.265	0.850–1.882	0.247	1.741	1.160–2.612	0.007*
P14+/P53-	reference			Reference		
P14-/P53+	1.532	0.633–3.712	0.344	1.832	0.552–6.081	0.323
P14-/P53-	reference			Reference		

* Statistically significant p<0.05

† Cox proportional hazard regression

#### ii) Effect of combining P53 and P16 expression

**Fig 4:**
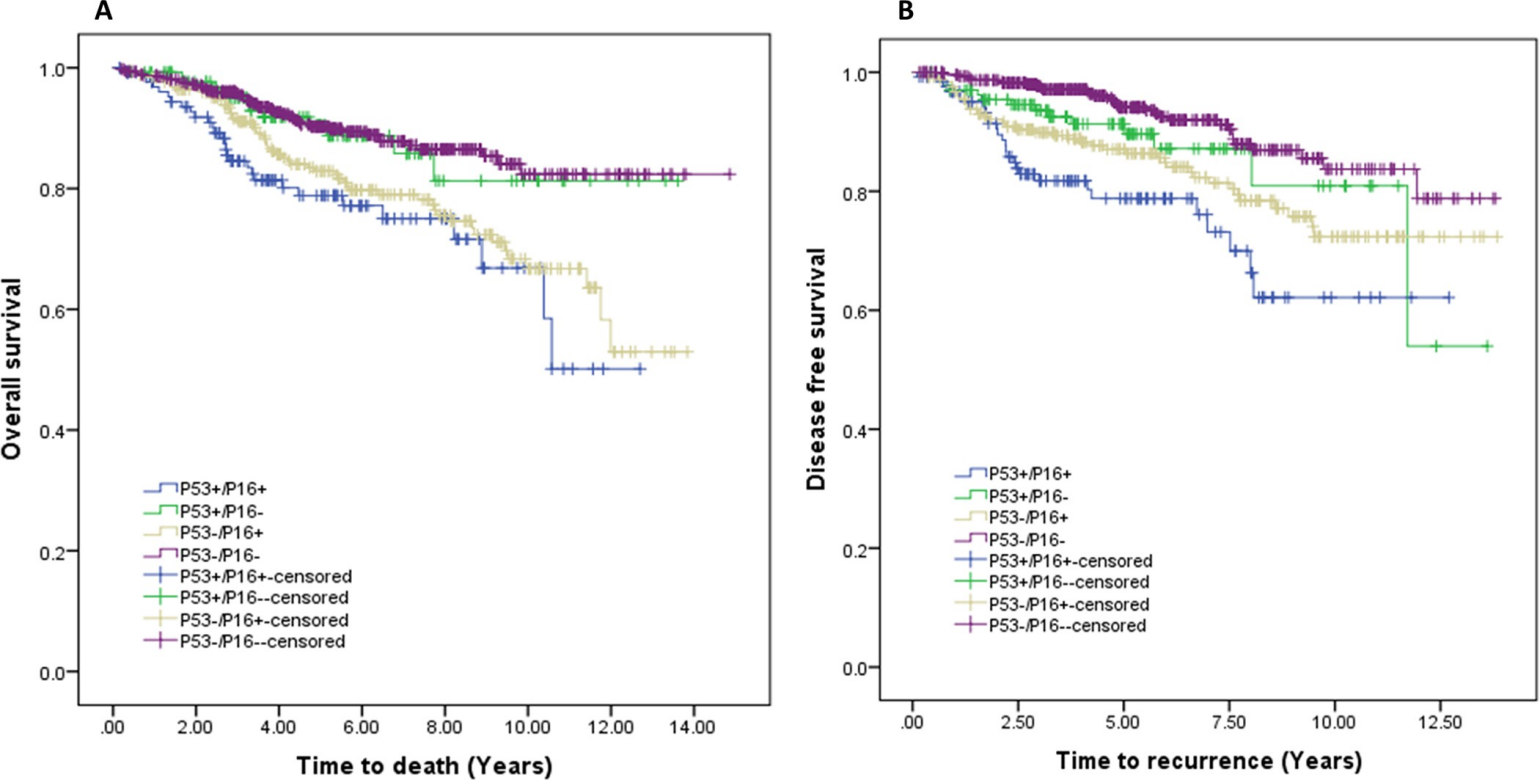
Survival probability based on joint effect of P53 and p16 expression. **A:** Overall survival probability, P53+/P16+ VS. P53-/P16- (p = 0.000), P53+/P16+ vs. P53+/P16- (p = 0.008), P53+/P16- VS. P53-/P16+ (p = 0.049) and P53-/P16+ vs. P53-/P16- (p = 0.000) showed significant differences on overall survival. The worst outcome was seen in patients P53+/P16+ tumours.**B**: Disease free survival, P53+/P16+ VS. P53-/P16- (p = 0.000), P53+/P16+ vs. P53+/P16- (p = 0.017) and P53-/P16+ vs. P53-/P16- (p = 0.000) showed significant differences in DFS. The worst outcome was seen in patients with P53+/P16+ tumours.

The effects of combining P53 and P16 expression on patient’s outcome were analysed using univariable models. Combination of P53 and P16 showed significant differences in patient’s overall survival and disease-free survival (Fig 4). The worst outcome was seen in patients with P53+/P16+ tumours. Relative to patients with P53 and P16 negative cancers, patients with breast cancers displaying strong P53 and P16 expressions had an adjusted 4-fold increased risk of having disease recurrence (HR = 3.748, 95% CI: 2.251–6.239) and 3-fold increased risk of all-cause related death (HR = 2.632, 95% CI: 1.656–4.183) (Table 5).

**Table 5:** Association of P53 and p16 status with overall and disease-free survival.

	Time to death			Time to recurrence		
	HR	95% CI	p-value†	HR	95% CI	p-value†
a) Joint effect						
P53+/P16+	2.632	1.656–4.183	<0.0001*	3.748	2.251–6.239	<0.0001*
P53+/P16-	1.090	0.589–2.015	0.784	1.706	0.912–3.190	0.094
P53-/P16+	2.031	1.379–2.991	<0.0001*	2.244	1.424–3.536	<0.0001*
P53-/P16-	reference			reference		
b) Stratified effect						
P53+/P16+	2.364	1.228–4.551	0.010*	2.158	1.130–4.121	0.020*
P53+/P16-	Reference			Reference		
P53-/P16+	2.029	1.378–2.989	<0.0001*	2.220	1.408–3.499	0.001*
P53-/P16-	Reference			Reference		

* Statistically significant p<0.05

† Cox proportional hazard regression

## Discussion

The importance of cellular senescence pathway in tumourigenesis has emerged as a potent protective response to oncogenic events. The activation of the pathway occurred early in the development of breast cancer. P53, DEC1 and DCR2 appear to increase in expression in the more advanced stages of the development of breast cancer, peaking at the premalignant DCIS stage, followed by a slight drop or plateauing in the invasive lesion. A similar expression pattern has been seen for all senescence-associated markers studied in our cohort of breast cancer progression. These observations may be explained by the tumour suppressive function of senescence markers reacting to cellular stress signals such as abnormal proliferation or DNA damage. Therefore, the tumour suppressor upregulation is a natural response to counteract the abnormal growth. However, invasive cancer may be formed after the cells surpass the cell cycle regulatory mechanisms when mutations occur in the tumour suppressor gene itself. It is reported that mutations of P53 are one of the most common known genetic alterations in human cancer [13, 17–19]. More than 75% of the mutations do not affect the translation of P53 protein, but mutant P53 protein loses its wild-type functions [20]. Mutant P53 is more stable compared to the short-lived wild-type P53 [21]. The long presence of mutant P53 itself may have an oncogenic effect on the cells [22]. However, it remains unknown whether the upregulation of P53 in this study is representative of the wild-type or mutant P53 protein.

Unlike P53, the importance of de novo markers is less well established in breast cancer. The overexpression of DEC1 increases from normal to ADH to DCIS but decreases from DCIS to IDC for both nuclear and cytoplasmic expression; however, in each tissue core the percentage of staining is small. Similarly, studies by other groups found an increase in the expression of DEC1 during progression from normal to in situ and invasive breast carcinoma [23]. This is in common with the finding of high expression of DEC1 in tumour tissues when compared to normal tissues in human kidney and lung [24]. These finding suggest that DEC1 may contribute to breast cancer progression to the invasive phenotype. It may additionally have a role in the stroma since expression was frequently observed in the adjacent background particularly in fibroblasts and white blood cells.

We found that DCR2 is highly expressed in normal breast tissue (60.4%), whereas expression of DCR2 occurs in a high proportion of cases, accounting for 93.9% of IDC. It was reported that senescent cells expressing high DCR2 protein show a less aggressive proliferation index, whereas loss of DCR2 expression in tumour cells is associated with high proliferation rate [2]. Overexpressed DCR2 is also seen in other human malignancies such as colon cancer and lung cancer [25, 26]. In contrast, Gottwald et al. reported DCR2 protein is less common in endometrioid adenocarcinoma compared to normal endometrium [27]. Physiologically, DCR2 is frequently expressed in normal tissues, but often silenced by hypermethylation in a wide range tumour cells including breast cancer, cervical cancer, malignant mesothelioma, neuroblastoma and prostate cancer [28].

The clinical significance of senescence protein in breast cancer was determined by assessing its relation to the existing standard prognostic factors. We found P53 expression was highly associated with the clinical and pathological parameters of breast cancer patients. Our study showed absence of P53 was significantly associated with favourable prognostic factors such as moderate differentiation, early stage tumour, absence of lymphovascular invasion, positive ER and PR status as well as negative HER2. To our knowledge, such strong association has not been reported in previous breast cancer studies. Only a few studies reported relationship between P53 protein expression with favourable clinicopathological parameters (young age [29]), while most studies have shown relationship between P53 protein expression with unfavourable indicators (large tumour size, high grade tumour, advanced tumour stage, negative hormone receptor status, lymph node invasion [30–33]). Silvestrini et al. confirmed that tumors overexpressing P53 are most frequently negative for estrogen receptor and that they tend to be large [34]. The relationship between absence of P53 and good prognostic indicators may be due to wild-type P53 having a short half-life of 5–20 minutes in most cells and is thus usually undetectable by standard immunohistochemical staining [21]. On the other hand, mutant P53 is more stable and accumulates in the nucleus due to elongated half-life or by binding with other oncogene proteins. An elevated level of mutated P53 does not give protection to the cellular integrity, and thus is directly linked to unfavourable prognostic factors. This finding may suggest that the upregulation of P53 is associated with mutant P53 protein. Nevertheless, there is no conclusive evidence that positivity in IHC staining is a result of P53 mutations, thus further analysis is required.

Interestingly, we found overexpressed DCR2 protein is associated with unfavourable prognostic factors such as poor tumour differentiation, ER and PR negativity and HER2 positivity. This association may reflect the activation of senescence pathway but somehow favour the tumorigenesis. It is reported that cellular senescence may activate the protective effect at the early stage of carcinogenesis but then evolve to stimulate neoplastic growth in later progression. In addition, the negative influence of overexpressed DCR2 may be due to its anti-apoptotic effects [35, 36]. DCR2 would compete with the death of TRAIL receptors to bind with Apo2L but its ligand formation prevents apoptosis thus favouring tumour progression. It is not fully established whether activation of DCR2 protein would favour senescence or apoptosis. The clinical significance of DEC1 in association with the well-established standard prognostic markers is not statistically significant.

Extensive biological and clinical studies have reported P53 may have promising value in predicting the outcome of a specific cancer type. However, it is not yet widely accepted and used in the clinical setting. The College of American Pathologist Consensus Statement has categorised P53 into category II of prognostic factor of breast cancer that remains to be validated through statistically robust studies [37]. There is a need to report a large group study that can give statistically strong evidence. The importance of P53 overexpression in predicting worse survival outcome has been reported in several small group breast cancer studies [38–40]. In the present study, we found P53 protein overexpression is correlated with increased risk in developing the disease later but not with the patient survival. Similar finding had been shown previously by others which found that overexpression of P53 has worse outcome for disease-free survival and the overall survival [11, 13, 41, 42]. The prognostic value of P53 overexpression has been associated with clinical breast cancer subtype of luminal A [43], node-positive [44] and metastatic breast cancer [45]. Moreover, other researchers have shown that P53 overexpression is a specific poor prognostic factor for lymph node metastasis, triple negative and HER2+/P53+ breast cancer [33, 46, 47]. P53 overexpression is reported to be related to aggressive tumour thus possibly explaining its association with poor prognosis. Furthermore, the association of P53 gene mutation with P53 protein overexpression may contribute to the abrogation of the suppressor pathway thus leading to aggressive tumour behaviour. Additionally, the poor prognosis may be possibly explained by its association with endocrine therapy resistance and reduced sensitivity to chemotherapy [43, 48]. DEC1 and DCR2 do not appear to be important prognostic factor for breast cancer patients in our study.

A single prognostic factor is no longer sufficient in treatment decision making. The conventional approach of ‘one size fits all’ has shifted to personalized treatment based on molecular classification of breast tumour. Currently, there is increasing use of two or more biomarkers in the assessment of patient prognosis and response to treatment [49]. The importance of the combined effect of P53 with P14 and P16 (data regarding P14 and P16 has been published previously [10]) as biomarkers is highlighted in this article. We have shown in this study that combination of P53 and p16 expression results in worse clinical outcome for overall and disease-free survival. However, the combination of p14 and P53 has a significant association with increased risk of disease recurrence in breast cancer patients. The combined effect has greater effect than single effect. It is important to identify factors that may modify the effectiveness of therapy because these may provide new targets for modification of drug resistance.

## Conclusion

In conclusion, defining senescence biomarker profiles in the earlier progression of invasive breast carcinoma may be potentially predictive of breast cancer patient’s survival. We have shown that the expression of these markers plateau or slightly decrease from premalignant to malignant lesions, consistent with tumour evasion of the senescence pathway. The overexpression of P53 can be used to predict poor disease-free survival and combination of P53 with P16 may provide more useful clinical information on the breast cancer survival outcome rather P53 expression alone.

## Supporting information

S1 Dataset De-identified minimal data set. (SAV)
